# Supplementary material for: Boosting diabetes and pre-diabetes detection in rural Ghana
Source: F1000Res. 2019 Aug 27;8:289. Originally published 2019 Mar 14. [Version 2] doi: 10.12688/f1000research.18497.2 (PMC6720030; doi:10.12688/f1000research.18497.2)
Supplement: Peer review history

# Peer review: Reviewer response for version 2

**DOI:** 10.5256/f1000research.21827.r53050

**Published:** 2019-09-02

**Authors:** Juan Salazar

**Affiliations:** 1 Endocrine and Metabolic Diseases Research Center, School of Medicine, University of Zulia, Maracaibo, Venezuela

The authors have taken into account the recommendations in this new version, especially regarding the organization and limitations of the study, which shows a promising approach in low-income regions.

I confirm that I have read this submission and believe that I have an appropriate level of expertise to confirm that it is of an acceptable scientific standard.

# Peer review: Reviewer response for version 2

**DOI:** 10.5256/f1000research.21827.r53049

**Published:** 2019-08-28

**Authors:** Frank Peter Schelp

**Affiliations:** 1 Faculty of Public Health, Khon Kaen University, Khon Kaen, Thailand

This is a well thought, adequate attempt, using appropriate technology, to explore the feasibility to install secondary preventive measures alongside type 2 diabetes mellitus in rural areas of  Ghana. There are minor misprints within the tables. Please check for capital letters and delete repeated words.

I confirm that I have read this submission and believe that I have an appropriate level of expertise to confirm that it is of an acceptable scientific standard.

# Peer review: Reviewer response for version 1

**DOI:** 10.5256/f1000research.20239.r50381

**Published:** 2019-07-01

**Authors:** Frank Peter Schelp

**Affiliations:** 1 Faculty of Public Health, Khon Kaen University, Khon Kaen, Thailand

From a historical point it is of interest, that this publication is submitted from authors from Ghana. That is remarkable, as the topic deals with diabetes mellitus, a disease, mainly due to overnutrition and obesity, from a country, where Cecily Delphine Williams once worked and published in 1935 the key paper about ‘Kwashiorkor’, one of the most severe and deadly conditions due to undernutrition. This reflects the drastic change of public health problems over time, due to the ‘epidemiological transition’.

 The manuscript is also remarkable, in that it shows how possibly a community approach, might help to control diabetes mellitus. This is within the spirit of ‘primary health care’, applied to a non-communicable disease.

- The authors report how community health nurses, equipped with inexpensive, appropriate technical innovations, measure capillary blood glucose, assess the BMI, blood pressure and collect information about additional diseases. For the study qualitative and quantitative research methods were applied. The authors repeatedly point out, that one of the major intentions of the project was to study the possibility and feasibility of using community health nurses for working against diabetes mellitus. The paper, with major improvements, will be acceptable as far as solely the qualitative approach is followed in trying to meet the objective. From the point of view of epidemiology however, the quantitative approach is inadequate. To use the term ‘screening’ is seriously misleading (please see page 257 and 259 of Porta, 2014 ^1^). To ‘introduce a piloted bias’ is an innovative euphemism by the authors and it seems, that they have their own doubts about a meaningful quantitative assessment based on groups of participants being too small and too heterogeneous.
- The authors might consider to change the now misleading title, to one which hints towards the objective of the publication more clearly.
- The last paragraph of the introduction should be revised and should mention the aims of conducting some sort of quality study.
- The methods and results sections might be combined. More inside information about the communities would be of benefit. Briefly mentioning the assumed knowledge of the participants about diabetes and the attitude towards the disease could help to estimate the usefulness of intervention. A short explanation how the community health worker fit into the overall health care for communities at the periphery of the overall health care system would be worthwhile as well. The section could be structured along the methods applied and for each method the usefulness, the acceptance of the participants within the communities, and the experience of the field worker could be described.
- In the discussion the overall experience gained of the project should be summarized in view of how an attempt to control diabetes mellitus in the communities in screening for new cases and caring for diseased patients would be helpful for further investigations and will be informative for local as well as national health workers and administrators. Additional points of interest would be to briefly mention the general dietary intake of the population and how this relates to overweight and diabetes. Last but not least, the question might be addressed on whether cut-off points for ‘normal’ and ‘not-normal’ glucose levels, as suggested internationally, are meaningful for Ghana, and what is the reaction of the authors to the recent suggestion to disregard ‘prediabetes’ as a diagnosis (see Piller, 2019 ^2^).

I confirm that I have read this submission and believe that I have an appropriate level of expertise to state that I do not consider it to be of an acceptable scientific standard, for reasons outlined above.

## References

[1] : A Dictionary of Epidemiology, Sixth Edition. *Oxford University Press*.2014; Reference source

[2] : Dubious diagnosis. *Science*.2019;363(6431) : 10.1126/science.363.6431.1026 1026-1031 10.1126/science.363.6431.1026 30846581

# Response

**Published:** 2019-08-20

**Authors:** Alessandro Crimi

**Affiliations:** African Institute for Mathematical Sciences, Ghana

**We have listed here our answers starting with an «(A)».**

- The paper, with major improvements, will be acceptable as far as solely the qualitative approach is followed in trying to meet the objective. From the point of view of epidemiology however, the quantitative approach is inadequate. To use the term ‘screening’ is seriously misleading (please see page 257 and 259 of Porta, 2014 ^1^). **(A)** *We agree with the reviewer that given our context and sample size the term «detection» could be more suitable.*
- To ‘introduce a piloted bias’ is an innovative euphemism by the authors and it seems, that they have their own doubts about a meaningful quantitative assessment based on groups of participants being too small and too heterogeneous. **(A)** *The authors are sure about the approach considering the goal of increasing early detection and we trust that «our introduced piloted bias» for the purpose is sensible. However, we are perfectly aware that due to funding we are using a small sample size; this is reported more strongly in the limitation of the study.*
- The last paragraph of the introduction should be revised and should mention the aims of conducting some sort of quality study. **(A)** *We agree with the reviewer, and we have softened the part regarding «novel determinants» which is not the main aim of the study.*
- The methods and results sections might be combined. More inside information about the communities would be of benefit. Briefly mentioning the assumed knowledge of the participants about diabetes and the attitude towards the disease could help to estimate the usefulness of intervention. A short explanation how the community health worker fit into the overall health care for communities at the periphery of the overall health care system would be worthwhile as well. The section could be structured along the methods applied and for each method the usefulness, the acceptance of the participants within the communities, and the experience of the field worker could be described. **(A)** *The idea of merging methods and results could be unproductive, and actually against the comments of the other reviewer which is asking even more structure. The additional information might be useful and we acknowledge that maybe we considered it as obvious. More specifically, the communities in exam are used to interact with community nurses and there is a high level of trust. The population is also generally aware of the importance of early diagnosis, though very often they restrain from it due to the related costs not covered by the normal insurance.*
- In the discussion the overall experience gained of the project should be summarized in view of how an attempt to control diabetes mellitus in the communities in screening for new cases and caring for diseased patients would be helpful for further investigations and will be informative for local as well as national health workers and administrators. Additional points of interest would be to briefly mention the general dietary intake of the population and how this relates to overweight and diabetes. Last but not least, the question might be addressed on whether cut-off points for ‘normal’ and ‘not-normal’ glucose levels, as suggested internationally, are meaningful for Ghana, and what is the reaction of the authors to the recent suggestion to disregard ‘prediabetes’ as a diagnosis (see Piller, 2019 ^2^).

1. Porta M: A Dictionary of Epidemiology, Sixth Edition. *Oxford University Press*. 2014.

2. Piller C: Dubious diagnosis. *Science*. 2019; **363** (6431): 1026-1031

**(A)** *The paper was submitted in early 2018, when the «opinion» of C. Piller was published at the beginning of 2019. We share the concerns in Europe and US about the abuse of medication for pre-diabetes. This is however, out of place especially considering the context of rural Ghana. Nevertheless, we thank the reviewer for pointing this out. We agree that we should avoid medications for pre-diabetes, while we should promote more awareness on inexpensive physical activities as jogging. Most of the points raised by the reviewers can be addressed by further investigation on physical activities and social marketing campaigns on physical activities.*

# Peer review: Reviewer response for version 1

**DOI:** 10.5256/f1000research.20239.r49360

**Published:** 2019-06-25

**Authors:** Juan Salazar

**Affiliations:** 1 Endocrine and Metabolic Diseases Research Center, School of Medicine, University of Zulia, Maracaibo, Venezuela

This is a report by Nyarko *et al.*, who propose a novel screening strategy for diabetes mellitus in a rural population of Ghana, Africa. An aspect of regional epidemiological relevance that could be reproduced in other geographic locations with similar socioeconomic characteristics.

**Abstract:**

- Introduction: it is not necessary to define prediabetes in this line.
- In Methods, it should describe the type of study and sampling. How were those 101 subjects chosen?
- In Results, the sentence "The need for further campaigns on alcohol and physical activity has emerged, even in rural areas", must go in Conclusions not in Results.

**Introduction: **

- It is somewhat extensive, paragraphs 2 and 3 could be more concise, emphasizing the justification for the design of this study.

**Materials and methods: **

- This section is somewhat disorganized, especially at the sample selection section. For this, it would be convenient to divide by subtitles according to the stages of the project, e.g.:

              - Study design and sample selection

               - Screening phase

               - Diabetes subjects selection phase

               - Clinical and capilary sugar evaluation

               - Definitions

               - Data analysis

               - Quantitative approach

               - Qualitative approach

- You must specify what type of sampling was used for both groups analyzed.
- Were the questions used by the mobile app validated or standardized, and by what method? There are some standardized questionnaires to assess the risk of diabetes in "healthy" subjects, such as the Diabetes Risk Test (ADA) and FINDRISK test. Why were some of these not used?

**Results:**

- There are some characteristics that must be specified in a table or figure:

1. In the USP group, how many subjects were prediabetic and diabetic according to the method used?
2. What were the determinants or risk factors most often evidenced in this group?
3. In the CDP group, how many subjects received pharmacological treatment? What were the most commonly used drugs? What were the factors or determinants of risk in control disease?
4. Although some comparisons are described in the text, as a screening study, some characteristics must also be specified in a table or figure to evaluate the proportions comparatively. In table 2 it is important to include the variables related to psychobiological habits, family and personal history of cardiovascular diseases and their risk factors.

**​​​Discussion:**

- It would also be advisable to organize this section (without the need to place subtitles) to make it easier to read.
- What is known about this topic? Especially in your region. Main findings of the study.
- What does this study add?
- Limitations: Mention in detail in this section.

I confirm that I have read this submission and believe that I have an appropriate level of expertise to confirm that it is of an acceptable scientific standard, however I have significant reservations, as outlined above.

# Response

**Published:** 2019-08-20

**Authors:** Alessandro Crimi

**Affiliations:** African Institute for Mathematical Sciences, Ghana

**We have listed here our answers starting with an «(A)».**

**Abstract:**

- Introduction: it is not necessary to define prediabetes in this line. **(A)** *We have removed the sentence.*
- In Methods, it should describe the type of study and sampling. How were those 101 subjects chosen? **(A)** *As we explained later in the paper, subjects were chosen through snowball sampling.*
- In Results, the sentence "The need for further campaigns on alcohol and physical activity has emerged, even in rural areas", must go in Conclusions not in Results. **(A)** *We agree with the reviewer.*

**Introduction:**

- It is somewhat extensive, paragraphs 2 and 3 could be more concise, emphasizing the justification for the design of this study.

**(A)** *We relatively disagree with the reviewer. Paragraph 2 is about Diabetes in low and middle income countries, while Paragraph 3 is about Ghana. We do believe all information reported is currently relevant. Otherwise we ask the reviewer to point out which sentences are not relevant.*

**Materials and methods:**

- This section is somewhat disorganized, especially at the sample selection section. For this, it would be convenient to divide by subtitles according to the stages of the project, e.g.:

              - Study design and sample selection

              - Screening phase

              - Diabetes subjects selection phase

              - Clinical and capilary sugar evaluation

              - Definitions

              - Data analysis

              - Quantitative approach

              - Qualitative approach

***(A)*** *We have inserted further subtitles suggested by the reviewer and shuffled some sentences to fit the structure.*

- You must specify what type of sampling was used for both groups analyzed. **(A)** *Snow-balling sampling entrusted to the community nurses was used.*
- Were the questions used by the mobile app validated or standardized, and by what method? There are some standardized questionnaires to assess the risk of diabetes in "healthy" subjects, such as the Diabetes Risk Test (ADA) and FINDRISK test. Why were some of these not used? **(A)** *The authors are aware of existing standardized questionnaires. However, they are relatively simplistic and comprising basic information not specific for the case in exam. Indeed most of the questions of the standardized questionnaires are included in our ad-hoc questionnaire. We envision our questionnaire as an expansion of the standardized questionnaire. Nevertheless, we are including this clarification.*

**Results:**

- There are some characteristics that must be specified in a table or figure:

1. In the USP group, how many subjects were prediabetic and diabetic according to the method used? **(A)** *This is information is already included in the paper. This is even mentioned in the abstract.*
2. What were the determinants or risk factors most often evidenced in this group? **(A)** *Unfortunately we have not found novel strong dietary determinants. The existence of relatives with diabetes for the pre-diabetic group is not a novel discovery. Our contribution is that it is possible to perform early detection of diabetes and pre-diabetes and that there is still a general misconception of what «physical activity» is.*
3. In the CDP group, how many subjects received pharmacological treatment? What were the most commonly used drugs? What were the factors or determinants of risk in control disease? **(A)** *All subjects in the CDP group were following pharmacological treatment, but we do not know the details of this.*
4. Although some comparisons are described in the text, as a screening study, some characteristics must also be specified in a table or figure to evaluate the proportions comparatively. In table 2 it is important to include the variables related to psychobiological habits, family and personal history of cardiovascular diseases and their risk factors. **(A)** *We are not in possession of detailed information about cardiovascular diseases apart from hypertension. However, if not reported most likely there is no past medical history of cardiovascular diseases for the subjects in exam. The study focused on the early detection of diabetes and pre-diabetes, we will acknowledge in the discussion section that deeper investigation on cardiovascular disease is missing and represents a limitation.​​​*

**Discussion:**

- It would also be advisable to organize this section (without the need to place subtitles) to make it easier to read.
- What is known about this topic? Especially in your region. Main findings of the study.
- What does this study add?
- Limitations: Mention in detail in this section.

**(A)** *We have restructured the discussion section, particularly adding a paragraph on «what is known» and a paragraph about «limitations».*
